# Television viewing and food intake during television viewing in normal-weight, overweight and obese 9- to 11-year-old Canadian children: a cross-sectional analysis

**DOI:** 10.1017/jns.2014.72

**Published:** 2015-02-27

**Authors:** Michael M. Borghese, Mark S. Tremblay, Genevieve Leduc, Charles Boyer, Priscilla Bélanger, Allana G. LeBlanc, Claire Francis, Jean-Philippe Chaput

**Affiliations:** 1 Children's Hospital of Eastern Ontario Research Institute, Healthy Active Living and Obesity Research Group, Ottawa, ON, Canada; 2 School of Human Kinetics, Faculty of Health Sciences, University of Ottawa, Ottawa, ON, Canada; 3 Department of Pediatrics, Faculty of Medicine, University of Ottawa, Ottawa, ON, Canada; 4 Institute of Population Health, University of Ottawa, Ottawa, ON, Canada

**Keywords:** Childhood obesity, Sedentary behaviour, Screen time, Food intake, CDC, Centers for Disease Control and Prevention, ISCOLE, International Study of Childhood Obesity, Lifestyle and the Environment, MVPA, moderate-to-vigorous physical activity

## Abstract

It is unclear if children of different weight status differ in their nutritional habits while watching television. The objective of the present paper was to determine if children who are overweight or obese differ in their frequency of consumption of six food items while watching television compared with their normal-weight counterparts. A cross-sectional study of 550 children (57·1 % female; mean age = 10 years) from Ottawa, Canada was conducted. Children's weight status was categorised using the Centers for Disease Control and Prevention cut-points. Questionnaires were used to determine the number of hours of television watching per day and the frequency of consumption of six types of foods while watching television. Overweight/obese children watched more television per day than normal-weight children (3·3 *v.* 2·7 h, respectively; *P* = 0·001). Obese children consumed fast food and fruits/vegetables more frequently while watching television than normal-weight or overweight children (*P* < 0·05). Children who watched more than 4 h of television per d had higher odds (OR 3·21; 95% CI 1·14, 9·03; *P* = 0·03) of being obese, independent of several covariates, but not independent of moderate-to-vigorous physical activity. The finding that both television watching and the frequency of consumption of some food items during television watching are higher in children who are obese is concerning. While the nature of the present study does not allow for the determination of causal pathways, future research should investigate these weight-status differences to identify potential areas of intervention.

Childhood obesity is an important public health concern and nearly one-third of Canadian children are considered overweight or obese^(^^1^^)^. Television viewing has been identified as an important behaviour associated with overweight and obesity in children^(^^2^^,^^3^^)^. High television viewing time^(^^4^^)^ has been shown to be positively associated with childhood obesity^(^^5^^–^^7^^)^. Historically, the association between television viewing and obesity in children has been thought to be driven by the displacement of moderate-to-vigorous physical activity (MVPA) with increased television viewing. However, there is evidence to suggest that the displacement hypothesis may not be the most likely mechanism driving this relationship^(^^8^^–^^11^^)^.

Another potential explanation for the association between television viewing and obesity is the role of food intake. Television viewing is associated with an overconsumption of food in the current obesogenic environment^(^^12^^)^. Specifically, high television viewing time has been reported to be inversely associated with fruit and vegetable consumption, and positively associated with consumption of energy-dense snacks and drinks, fast food and total energy intake^(^^13^^)^. However, the evidence for the role of food intake, specifically while watching television, in the association between television viewing and obesity is limited. The observation of increased food intake while watching television is thought to be driven by several distraction mechanisms and habituation to satiety cues^(^^14^^–^^18^^)^. Furthermore, television advertising can prompt consumption during viewing and may lead to unhealthy food choices^(^^3^^)^. Van den Bulck & Van Mierlo^(^^19^^)^ showed that for each 1 h increase in television viewing was associated with consumption of an additional 156 kcal/d (653 kJ/d) in youth. However, the authors examined only absolute energy intake, not food types, and did not differentiate between children of different weight status. It has been shown that children who are obese are more strongly affected by television advertisements, with respect to their food intake, as compared with children who are normal weight or overweight^(^^20^^)^. However, there is currently a lack of evidence on how children who are overweight/obese or of normal weight may differ in the frequency of consumption of food items in front of the television^(^^21^^)^. This is especially salient given the conclusion of a recent review by Mattes^(^^22^^)^, which suggests that ingestive frequency has a greater impact on energy balance than portion size. As children can consume 19–26 % of their daily energy intake in front of the television^(^^19^^,^^23^^)^, further research into the types of food that children consume in front of the television, as well as whether or not the frequency of consumption differs between children of different weight status, is needed.

As suggested above, there is limited evidence examining how the frequency of consumption of food items while watching television differs between normal-weight, overweight and obese children. The objective of the present paper was to compare children of different weight-status groups in the frequency of consumption of food items while watching television. This analysis is novel as it considers the role of consumption of food items specifically while watching television. It is hypothesised that children who are overweight or obese would: (1) watch more television each day; (2) report consuming ‘unhealthy’ foods more frequently while watching television; and (3) report consuming fruits and vegetables less frequently while watching television than their normal-weight counterparts.

## Methods

### Participants

The International Study of Childhood Obesity, Lifestyle and the Environment (ISCOLE) is a multi-national, cross-sectional study conducted in twelve countries. Data from the National Health and Nutrition Examination Survey (NHANES) 2005/2006 informed an *a priori* power calculation which indicated that a sample size of 500 participants from each of the twelve international sites would allow for sufficient statistical power for the primary research question of ISCOLE. Details pertaining to the study design and methods, including further information on sample size justification, can be found elsewhere^(^^24^^)^. Analyses herein include data from the Canadian site of ISCOLE. Data were collected in schools on 567 children (57·1 % female; 9–11 years of age) from the Ottawa area between September 2012 and May 2013. Consent forms were distributed to 1161 children, of whom 597 participated in the study (51·4 %) and 567 completed the study (48·8 % of those contacted; 95·0 % of those who participated). Schools were stratified into four groups: English public (*n* 393; 69·3 %); French public (*n* 60; 10·6 %); English Catholic (*n* 75; 13·2 %); and French Catholic (*n* 39; 6·8 %). This project was approved by the research ethics boards at the Children's Hospital of Eastern Ontario and the University of Ottawa, as well as by the participating school boards.

### Anthropometric measurements

Trained study staff collected anthropometric data and administered questionnaires in schools during school hours. Weight was measured to the nearest 0·1 kg using a portable Tanita Body Composition Analyser (SC-240; Tanita). Standing height and sitting height were measured to the nearest 0·1 cm using a SECA 213 portable stadiometer. Normal weight, overweight and obesity were defined as >5th but <85th, ≥85th but <95th, and ≥95th age- and sex-specific percentiles, respectively, as defined by the Centers for Disease Control and Prevention (CDC)^(^^25^^)^. CDC cut-points were used to define overweight and obesity to allow for comparison with much of the published literature. Children who were underweight (*n* 15), as well as children who did not provide a valid measure of weight (*n* 2), were excluded from the analysis; thus, data were analysed with 550 children.

### Television viewing and FFQ

During the school visit participants completed a diet and lifestyle questionnaire which included a self-reported measure of television viewing for each day as well as a specialised FFQ to assess the frequency of consumption of six food items while watching television^(^^24^^)^.

The screen-time questionnaire, derived from the US Youth Risk Behaviour Surveillance System (YRBSS)^(^^26^^)^, asked children how many hours of television they watch on a typical school day as well as on a typical weekend day. The television viewing time question derived from the YRBSS was shown to have adequate reliability (Spearman correlation 0·55–0·68) and validity (Spearman correlation 0·47)^(^^27^^)^. The response options included: no television watching, <1 h, 1 h, 2 h, 3 h, 4 h and ≥5 h of television per d. A weighted mean number of hours of television watching per day was calculated as follows:

hours of television watching per d = ((hours of television on weekdays × 5) + (hours of television on weekend days × 2))/7.

Self-report methods of quantifying screen time have been shown to have acceptable reliability and validity in children^(^^28^^)^.

The FFQ during television viewing was adapted from Van den Bulck & Van Mierlo^(^^19^^)^, and asked the participants how often they consumed five ‘unhealthy’ food items and one ‘healthy’ food item while watching television in a usual week. Unhealthy foods included: (1) potato chips or peanuts; (2) fried foods such as chicken wings, chicken fingers, French fries, etc.; (3) cookies, biscuits, chocolate or candy bars; (4) ice cream; and (5) fast foods such as pizza, hamburgers, etc. Healthy foods included fruits or vegetables. There were seven response options ranging from ‘never’ to ‘every day, more than once’.

### Covariates

Demographic questionnaires completed by parents were used to determine children's age (from date of birth reported by the parents), sex and ethnicity (white/Caucasian, African American, Asian, First Nations, East Indian, ‘don't know’, or ‘other’), along with total annual family income (eight options ranging from less than $14 999 to $140 000 or more), and the highest level of parental education (less than high school, some high school, high school diploma/General Education Development (GED), diploma or 1–3 years of college, bachelor's degree, graduate (master's or PhD)/professional degree). Biological maturity was estimated using the maturity offset method, which estimates an individual's age from peak height velocity^(^^29^^)^. Nightly sleep duration was assessed using the ActiGraph GT3X+ (ActiGraph Corp.); a detailed description of this novel algorithm developed for the ISCOLE study is available elsewhere^(^^24^^)^. MVPA was also assessed using the ActiGraph GT3X+ with cut-points developed by Evenson *et al.* (>2296 counts per min)^(^^30^^)^. Further details with respect to accelerometry data reduction procedures are available elsewhere^(^^24^^)^. These covariates were chosen because of their association with television viewing and/or food intake patterns in the literature.

### Statistical analysis

Non-parametric tests for significant differences were performed given the nominal or ordinal nature of the data. Kruskal–Wallis ANOVA was used to determine significant differences between weight-status groups in their television watching and the frequency of consumption of food items while watching television. When Kruskal–Wallis ANOVA was significant, Mann–Whitney *U* tests were used *post hoc* to determine specifically which weight-status groups differed in television viewing time per day or frequency of consumption of food items. Ordinal regression or general linear models, where appropriate, were used to provide parameter estimates and 95 % CI for the associations between weight-status groups and both television viewing and frequency of consumption of food items in front of the television that were found to be significant in the Kruskal–Wallis ANOVA. In ordinal regression, the link functions were tested for model fit using –2 log likelihood, χ^2^ goodness of fit, as well as the test of parallel lines. Variables were either positively skewed or normally distributed; thus, negative log-log or logit link functions were tested and the link function that had the best model fit was selected. Logistic regression was used to compute OR (and 95 % CI) and adjusted OR (after adjustment for covariates) for the odds of being overweight or obese with high or moderate television viewing or high consumption of food items found to be significantly different in the Kruskal–Wallis ANOVA. Children who watched more than 4 h of television per d or 2–4 h of television per d were compared with children who watched ≤ 2 h of television per d; this is consistent with definitions of high and low television viewing in the literature^(^^4^^,^^31^^)^. High and low frequency of consumption while watching television was defined as above or below the median response for each food item. For fast foods, responses ‘never’ and ‘less than once/week’ were merged to represent low frequency of consumption, while responses ‘once a week’ up to ‘every day, more than once’ were merged to represent high frequency of consumption. For fruits and vegetables, responses from ‘never’ to ‘2–4 d/week’ were merged to represent low frequency of consumption, while responses ‘5–6 d/week’ to ‘every day, more than once’ were merged to represent high frequency of consumption. Finally, the ‘frequency rate’ of consumption of food items during television watching was computed by dividing the frequency of consumption of the food item per week by the number of hours of television watched per day; only those foods found to be significantly different between weight-status groups in the Kruskal–Wallis ANOVA were included in this analysis. This analysis was undertaken to examine if the differences in frequency of consumption of a food item between weight-status groups was due to differences in the number of hours of television viewing. A two-tailed *P* value of less than 0·05 was the threshold to indicate statistical significance. All statistical analyses were performed using SPSS version 21 (IBM Corp.).

## Results

Participant and parent demographic information is provided in Table 1. A total of 550 participants (42·9 % male) provided complete data for the variables of interest and were included in the analysis. There were no statistically significant sex interactions between television viewing and the outcome variables; thus, data for both sexes were merged to maintain statistical power. The prevalence of overweight and obesity was 12·4 % (68/550) and 10·9 % (60/550), respectively, using CDC cut-points.

**Table 1.** Descriptive characteristics of Canadian children in the International Study of Childhood Obesity (ISCOLE) study (*n* 550) (Number of subjects and percentages; mean values and standard deviations; medians and interquartile ranges (IQR))

	*n*	%
Age (years)		
Mean		10·0
sd		0·04
Sex		
Male	236	42·9
Female	314	57·1
Ethnicity		
White/Caucasian	360	66·3
African American	15	2·8
Asian	56	10·3
First Nations	2	0·4
East Indian	5	0·9
Don't know	1	0·2
Other	104	19·2
Maturity offset		
Median		−1·88
IQR		−2·74, –1·20
BMI category – CDC*		
Normal weight	422	76·7
Overweight	68	12·4
Obese	60	10·9
Television watching (h/d)		
<2	456	82·9
2–4	54	9·8
>4	40	7·3
Annual household income ($)		
<14 999	16	3
15 000–29 999	32	6·1
30 000–39 999	15	2·8
40 000–59 999	36	6·8
60 000–89 999	72	13·6
90 000–109 999	77	14·6
110 000–139 999	78	14·8
≥140 000	202	38·3
Highest level of parental education		
Less than high school	2	0·4
Some high school	9	1·7
High school diploma/GED	35	6·4
Diploma or 1–3 years of college	110	20·3
Bachelor's degree	169	31·1
Graduate (master's or PhD)/professional degree	218	40·1
Daily MVPA (min)		
Mean	58·9	
sd	19·2	
Nightly sleep duration (min)		
Mean	544·4	
sd	50·8	

CDC, Centers for Disease Control and Prevention; GED, General Education Development; MVPA, moderate-to-vigorous physical activity.

* Age- and sex-specific BMI-for-age percentiles^(^^25^^)^.

### Comparison of children by weight-status groups

Similar results were observed for weighted mean television watched per day and television watched on weekdays; however, results were not significant with television watching on weekend days. In all analyses using television viewing time the weighted mean number of hours of television viewing per day is reported to improve clarity. There was a significant main effect of weight status on television viewing, and children who were overweight or obese watched more television per day than those who were normal weight (Table 2). Additionally, in ordinal regression using a logit link function, being overweight was positively associated with reporting a higher television viewing time category on weekdays (β = 0·57; 95 % CI 0·11, 1·03; Wald χ^2^ = 6·1; *P* = 0·01), but not on weekend days (β = 0·31; 95 % CI –0·15, 0·76; Wald χ^2^ = 1·7; *P* = 0·19), as compared with being normal weight. Similarly, being obese was positively associated with reporting a higher television viewing time category on weekdays (β = 0·94; 95 % CI 0·45, 1·42; Wald χ^2^ = 14·4; *P* < 0·001), but not on weekend days (β = 0·48; 95 % CI –0·003, 0·95; Wald χ^2^ = 3·8; *P* = 0·052), as compared with being normal weight. Finally, using a general linear model, weight status was positively associated with higher television viewing time (*F* = 8·6; η^2^ = 0·03; *P* < 0·001), such that being overweight (β = 0·37; 95 % CI 0·002, 0·73; *P* = 0·049) or obese (β = 0·76; 95 % CI 0·37, 1·14; *P* < 0·001) was associated with a higher television viewing time, as compared with being normal weight.

**Table 2.** Hours of television viewing on both weekdays and weekends by BMI category, and differences between weight-status categories in Canadian children in the International Study of Childhood Obesity, Lifestyle and the Environment (ISCOLE) study (n 550, mean age 10 years) (Medians and interquartile ranges (IQR))

	BMI category‡							
	Normal weight (*n* 422)§		Overweight (*n* 68)§		Obese (*n* 60)§		Kruskal–Wallis ANOVA	
Hours of television viewing	Median	IQR	Median	IQR	Median	IQR	χ^2^	*P*
Weekdays	2·5	2·0–4·0	3·0†	2·0–4·0	3·0†	2·0–5·0	17·48	<0·01*
Weekends	4·0	3·0–4·3	4·0	4·0–5·0	4·0	2·3–6·0	4·29	0·12
Total week‖	2·7	2·0–3·9	3·3†	2·4–4·3	3·3†	2·3–5·2	14·78	0·01*

* Significant main effect (*P* < 0·05; Kruskal–Wallis ANOVA).

† Median was significantly different from that for the normal-weight group (*P* < 0·05; Mann–Whitney *U post hoc* test).

‡ Centers for Disease Control and Prevention age- and sex-specific BMI-for-age percentiles (http://www.cdc.gov/growthcharts/)^(^^25^^)^.

§ Response categories: 1 = no television watching, 2 = < 1 h, 3 = 1 h, 4 = 2 h, 5 = 3 h, 6 = 4 h, and 7 = 5 h or more of television per d.

‖ Weighted mean = ((number of hours on weekdays × 5) + (number of hours on weekends × 2))/7.

There was a significant main effect of weight status on the consumption of fast foods as well as fruits and vegetables while watching television. Obese children consumed fast food and fruits/vegetables more frequently while watching television than those who were normal weight or overweight (Table 3), but there were no differences in any of the other food items between weight-status groups. Additionally, in ordinal regression using a negative log-log link function, being overweight was not associated with self-reporting a higher frequency of consumption of fast foods in front of the television (β = –0·21; 95 % CI –0·56, 0·14; Wald χ^2^ = 1·4; *P* = 0·24). However, being obese was positively associated with self-reporting a higher frequency of consumption of fast foods in front of the television (β = 0·42; 95 % CI 0·10, 0·74; Wald χ^2^ = 6·5; *P* = 0·01), as compared with being normal weight. Similarly, in ordinal regression using a logit link function, being overweight was not associated with self-reporting a higher frequency of consumption of fruits or vegetables in front of the television (β = 0·13; 95 % CI –0·32, 0·56; Wald χ^2^ = 0·31; *P* = 0·58). However, being obese was positively associated with self-reporting a higher frequency of consumption of fruits and vegetables in front of the television (β = 0·80; 95 % CI 0·31, 1·28; Wald χ^2^ = 10·3; *P* = 0·001), as compared with being normal weight.

**Table 3.** Frequency of foods consumed while watching television per week by BMI category in Canadian children in the International Study of Childhood Obesity, Lifestyle and the Environment (ISCOLE) study (n 550, mean age 10 years) (Medians and interquartile ranges (IQR))

	BMI category§							
	Normal weight (*n* 422)‖		Overweight (*n* 68)‖		Obese (*n* 60)‖		Kruskal–Wallis ANOVA	
Frequency of consumption of food items	Median	IQR	Median	IQR	Median	IQR	χ^2^	P
Potato chips or peanuts	2	1–3	2	1–2·75	2	2–3	4·74	0·09
Fried foods¶	2	1–2	2	1–2·75	2	1–3	4·30	0·12
Snack foods**	2	1–3	2	1·25–3	2	2–3	0·14	0·94
Ice cream	2	1–3	2	1–2·75	2	1–3	1·14	0·57
Fast foods††	2	1–2	2	1–2·75	2†‡	2–3	6·14	0·04*
Fruits or vegetables	4	2–6	4	2–6	5·5†‡	4–7	10·95	<0·01*

* Significant main effect (*P* < 0·05; Kruskal–Wallis ANOVA).

† Median was significantly different from that for the normal-weight group (*P* < 0·05; Mann–Whitney *U post hoc* test).

‡ Median was significantly different from that for the overweight group (*P* < 0·05; Mann–Whitney *U post hoc* test).

§ Centers for Disease Control and Prevention age- and sex-specific BMI-for-age percentiles (http://www.cdc.gov/growthcharts/)^(^^25^^)^.

‖ Response categories: 1 = never; 2 = less than once per week; 3 = once per week; 4 = 2–4 d per week; 5 = 5–6 d per week; 6 = once per day, every day; 7 = every day, more than once.

¶ Fried food such as chicken wings, chicken fingers, French fries, etc.

** Snack foods such as cookies, biscuits, chocolate or candy bars.

†† Fast foods such as pizza, hamburgers, etc.

**Fig. 1.**
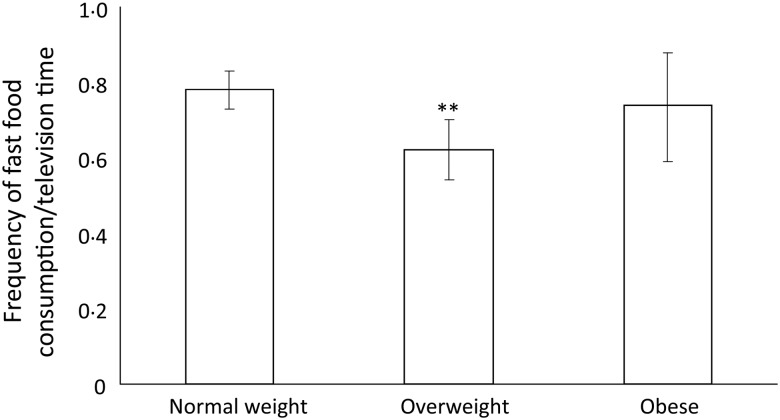
Frequency of fast food consumption while watching television per week divided by the number of hours of television watched per day by weight-status group in Canadian children in the International Study of Childhood Obesity, Lifestyle and the Environment (ISCOLE) study (*n* 550; mean age 10 years). Values are means, with 95 % CI represented by vertical bars. ** Mean value was significantly different from that of the normal-weight group (*P* = 0·01).

Finally, children who were normal weight ate fast food at a higher frequency rate while watching television than those who were overweight; however, normal-weight children did not differ from obese children in their frequency rate of fast food consumption during television watching (Fig. 1). There were no differences between weight-status groups for the frequency rate of consumption of fruits/vegetables while watching television (data not shown).

### Associations between television viewing and frequency of consumption of food items on weight status

Logistic regression analyses showed that children who watched more than 4 h of television per d had higher odds of being obese (Table 4), but did not have higher odds of being overweight (data not shown). This association remained statistically significant with the addition of age, sex, ethnicity, total annual family income, highest parental education level, and nightly sleep duration into the model. However, this association became non-significant with the addition of MVPA. Children who watched 2–4 h of television per d did not have higher odds of being overweight (data not shown) or obese (Table 4) in any of the models.

**Table 4.** Odds ratio of being obese if a child watches high amounts of television or eats fast food or fruits and vegetables more frequently while watching television in Canadian children in the International Study of Childhood Obesity, Lifestyle and the Environment (ISCOLE) study (*n* 550, mean age 10 years)* (Odds ratios and 95 % confidence intervals)

	Risk of being obese		
	OR	95 % CI	*P*
Watching 2–4 h of television per d†			
Model 1	1·32	0·53, 3·30	0·56
Model 2	0·66	0·21, 2·09	0·48
Model 3	0·59	0·18, 2·0	0·40
Watching >4 h of television per d†			
Model 1	6·00	2·83, 12·72	<0·01
Model 2	3·19	1·14, 8·87	0·03
Model 3	2·40	0·81, 7·05	0·11
Eating fast food while watching television ≥1 d per week‡			
Model 1	1·40	0·78, 2·53	0·25
Model 2	1·45	0·65, 3·20	0·36
Model 3	1·39	0·61, 3·20	0·44
Eating fruits and vegetables while watching television ≥4 d per week§			
Model 1	2·20	1·26, 3·83	<0·01
Model 2	1·58	0·77, 3·21	0·21
Model 3	2·02	0·95, 4·27	0·07

* Model 1, unadjusted. Model 2, adjusted for age, sex, ethnicity, biological maturity, total annual family income, highest parental education level, and nightly sleep duration. Model 3, additionally adjusted for moderate-to-vigorous physical activity.

† Reference group: watching ≤2 h of television per d.

‡ Reference group: eating fast food while watching television <1 d per week.

§ Reference group: eating fruits and vegetables while watching television <3 d per week.

Children who consumed fast food while watching television once or more per week did not have higher odds of being overweight or obese than those eating fast food less than once per week (Table 4); different cut-points around the median (i.e. 25th and 75th percentiles) for high/low frequency of consumption of fast food did not yield different results. Children who consumed fruits and vegetables while watching television on more than 4 d per week had higher odds of being obese, but not of being overweight as compared with children consuming fruits and vegetables less than 3 d per week (Table 4). This association did not hold with the addition of covariates. Once again, the choice of cut-point around the median (i.e. 25th and 75th percentiles) for high/low frequency of consumption of fruits and vegetables had no effect on the results.

## Discussion

In the current sample of 550 children, overweight/obese children watched more television per day than their normal-weight peers. Children who watched more than 4 h of television per d had higher odds of being obese, independent of several covariates; however, this relationship did not hold with the addition of MVPA. Finally, obese children consumed fast food and fruits/vegetables more frequently while watching television than the normal-weight or overweight children.

The finding that children who are overweight or obese watch more television than their normal-weight counterparts is consistent with the literature^(^^5^^,^^7^^)^. This finding is a cause for concern as children who are high users of television are likely to be high users as they age^(^^32^^)^, and television viewing in adults is associated in a dose–response manner with type 2 diabetes, cardiovascular events and all-cause mortality^(^^33^^)^.

Children who watched high amounts of television were found to have higher odds of being obese, independent of many covariates, but not independent of MVPA. The displacement hypothesis of television viewing and obesity suggests that higher television viewing time may reduce available discretionary time for MVPA in children, which has the potential to make an impact on energy balance. The current finding suggests that MVPA may confound the association between television viewing time and weight status. This finding is contrary to some studies using objective measures of physical activity in adolescents. Rey-López *et al.*^(^^34^^)^ found that boys, but not girls, who watched > 4 h of television per d had higher odds of being overweight, independent of MVPA. However, the study by Rey-López *et al.*^(^^34^^)^ did not adjust for any of the covariates considered in the analyses herein and residual confounding is a possibility. The same finding as that of Rey-López *et al.*^(^^34^^)^ was observed in the current analysis when only adjusting for MVPA (data not shown), illustrating the importance of controlling for a larger panel of potential confounders (if the study is sufficiently powered) when examining the relationship between television viewing and obesity in children. While there is more evidence to suggest that the association between television viewing and obesity in children is explained by alterations in dietary patterns than MVPA^(^^3^^)^, it is likely that both play a role in this association^(^^35^^)^. Few studies have considered the role of food intake while watching television on overweight and obesity in children. No study to date has done so with a large sample size while also considering many potentially confounding variables, factors that have led to mixed results in previous studies. Rey-López *et al.*^(^^34^^)^ found that adolescents who eat in front of the television once a week are at higher odds of being overweight after adjusting for MVPA^(^^34^^)^; however, the authors failed to consider the role of other confounding variables. Matheson *et al.*^(^^23^^)^ found that food intake while watching television was not associated with BMI in children^(^^23^^)^; however, the sample size was too small and heterogeneous (pooled from two studies, *n* 90 and *n* 124) to perform between-groups analyses or to adjust for several confounders.

Consumption of fast food is consistently associated with obesity^(^^13^^)^, and high amounts of television viewing are associated with obesity^(^^5^^–^^7^^)^ and increased fast food consumption^(^^36^^)^. Thus, the current finding that children who are obese consume more fast food while watching television may not come as a surprise, given the state of the literature. However, the finding that a high frequency of fast food consumption while watching television is not associated with higher odds of being overweight or obese in both unadjusted and adjusted models is intriguing. The relationship between fast food and obesity, at least as it relates to fast food eaten while watching television, may be better explained by reverse causality – obese children may consume more fast food, but consuming more fast food may not predict obesity. The cross-sectional nature of this analysis does not allow for the determination of causality, but future research should better delineate the association between fast food consumption during television viewing and obesity. Furthermore, as this analysis only considers the frequency of consumption of fast food during television watching, the absolute quantity and variation in quality cannot be determined.

Normal-weight children consumed fast food at a higher frequency rate while watching television than overweight children, but not obese children; there were also no differences between overweight and obese children. Given the descriptive results of the present study, this finding is not surprising – normal-weight and overweight children consume fast food in front of the television with a similar frequency; however, overweight children watch more television. Likewise, the magnitude of the difference in frequency of consumption of fast food while watching television between overweight and obese children is small, and they watch similar amounts of television. Thus, any differences in the frequency rate of consumption of fast food during television viewing between overweight and obese children may be too small to detect^(^^37^^)^. This is a novel analysis in the consideration of food intake during television viewing, as the frequency of consumption may be higher in obese children, but this is explained by higher television watching, not a higher frequency rate of fast food consumption. There is some evidence to suggest that obese children may have a heighted alertness to food-related cues associated with television viewing^(^^20^^,^^38^^)^; however, the present findings suggest that, at least in front of the television, obese children do not consume foods at a higher frequency rate.

The finding that children who are obese consume fruits and vegetables more frequently while watching television than their overweight and normal-weight counterparts is contrary to our hypothesis and most of the available evidence^(^^13^^,^^36^^,^^39^^,^^40^^)^. The median (interquartile range) of the frequency of consumption of fruits and vegetables is considerably higher than the other food items in the FFQ, especially for children who are obese. This may indicate a positive response or social desirability bias, as this differs from what is generally seen in the literature; however, this cannot be known with certainty. Children who are obese have higher daily energy requirements^(^^41^^)^ and so may consume many different types of foods more frequently than children who are overweight or normal weight; however, the absolute quantity and variation in quality of food intake cannot be determined from the present study. As this analysis does not consider the food that children eat in the absence of television watching, it is possible that obese children do not consume fruits and vegetables more frequently over the course of the entire day, but due to increased television watching more of this consumption might occur while watching television.

Furthermore, the co-consumption of other foods while consuming fruits and vegetables cannot be ruled out; if children who are obese consume more meals, which include fruits and vegetables, in front of the television this may lead to overconsumption of many food types which can promote a positive energy balance. In the current analysis, there was no effect of weight status on the frequency rate of consumption of fruits and vegetables while watching television, which suggests that obese children consume fruits and vegetables more frequently in front of the television simply because they watch more television. Also, the finding that fruit and vegetable consumption while watching television was associated with higher odds of being obese in the unadjusted model may provide evidence for this hypothesis. However, that the association became non-significant with the addition of covariates should remind us of the confounded nature of the association between food intake, television watching and obesity in children and of the many factors at play. Indeed, there are many factors that may explain the finding above and researchers in this field may wish to further examine the role of food intake while watching television and its impact on obesity, specifically considering the co-consumption of foods with fruits and vegetables, and evaluating the impact of eating in front of the television in the context of whole-day food intake.

There are several strengths of the present study, including the large sample of Canadian children, the robust data quality assurance procedures^(^^24^^)^ and the inclusion of many confounding variables which were not typically included in past research. Unique to this analysis is a novel covariate, total sleep time as measured by accelerometry, as well as biological maturity, which is an important consideration in such analyses. Finally, the use of an objective measure for the measurement of MVPA is an asset.

There are also several limitations to the current analysis. First, causality cannot be determined from cross-sectional data. Second, although we used a large sample of Canadian children, it is not known if the sample is nationally representative and therefore results may not be generalisable. Third, the present study did not differentiate between watching television alone or with peers, as well as the placement of televisions (i.e. in the bedroom), which has been associated with unfavourable body composition in itself^(^^42^^)^. Fourth, television viewing is notoriously difficult to measure, and better measures other than self-report exist^(^^43^^)^; however, the screen time frequency questionnaire used in the current analysis maximised feasibility for the given sample size and reduced participant burden. Fifth, this analysis does not take into account the effect of television watching on absolute food intake, or the effect of television advertisements on whole-day food intake; however, this was outside of the scope of the current research question. Finally, the FFQ is limited in its ability to assess food intake as it does not account for quantity or quality; however, that frequency alone may differ between weight-status groups in children is an important finding. Furthermore, there are no reliability or validity statistics available for the specialised FFQ; however, this tool was the only one of its kind to assess the frequency of consumption of food items during television watching.

Overall, these results are consistent with previous analyses which suggest that children who are overweight or obese watch more television than their normal-weight counterparts. A high frequency of consumption of foods in front of the television has the potential to make an impact on energy balance and ultimately body weight, especially in the paediatric obese population as these children may be more strongly affected by television advertisements^(^^20^^)^. From a public health perspective, it may be more feasible to focus our efforts on reductions in television viewing time than to suggest that children eat less frequently in front of the television, although both of these methods offer potential avenues for future intervention. To reduce health risk children should reduce both the amount of time they spend watching television as well as the frequency of eating in front of the television.

### Conclusion

High television watching was associated with obesity in the current sample of children aged 9–11 years independent of covariates, but not independent of MVPA. Obese children consumed fast food more frequently while watching television than normal-weight children; however, higher frequency of consumption of fast food was not associated with higher odds of being obese, which suggests that the association may be driven by the nutritional habits of obese children. Also, obese children consumed fruits and vegetables more frequently while watching television than normal-weight children. This finding is contrary to most of the literature and should be validated in future studies which examine the quantity and quality of food eaten, as well as the co-consumption of food eaten alongside fruits and vegetables while watching television. These findings suggest that both physical activity and diet play a role in the association between television viewing and obesity.
